# Urinary Metabolites as Predictors of Acute Mountain Sickness Severity

**DOI:** 10.3389/fphys.2021.709804

**Published:** 2021-09-13

**Authors:** Isaie Sibomana, Daniel P. Foose, Michael L. Raymer, Nicholas V. Reo, J. Philip Karl, Claire E. Berryman, Andrew J. Young, Stefan M. Pasiakos, Camilla A. Mauzy

**Affiliations:** ^1^Henry M. Jackson Foundation for the Advancement of Military Medicine, Bethesda, MD, United States; ^2^Air Force Research Laboratory, 711th Human Performance Wing, Wright-Patterson AFB, Dayton, OH, United States; ^3^Department of Computer Science and Engineering, Wright State University, Dayton, OH, United States; ^4^Boonshoft School of Medicine, Wright State University, Dayton, OH, United States; ^5^Military Nutrition Division, U.S. Army Research Institute of Environmental Medicine, Natick, MA, United States; ^6^Department of Nutrition, Food, and Exercise Sciences, Florida State University, Tallahassee, FL, United States; ^7^Oak Ridge Institute of Science and Education, Belcamp, MD, United States

**Keywords:** acute mountain sickness, susceptibility, altitude, hypoxia, NMR-based metabolomics, metabolite profiles, altitude sickness, urine

## Abstract

Individuals sojourning at high altitude (≥2,500m) often develop acute mountain sickness (AMS). However, substantial unexplained inter-individual variability in AMS severity exists. Untargeted metabolomics assays are increasingly used to identify novel biomarkers of susceptibility to illness, and to elucidate biological pathways linking environmental exposures to health outcomes. This study used untargeted nuclear magnetic resonance (NMR)-based metabolomics to identify urine metabolites associated with AMS severity during high altitude sojourn. Following a 21-day stay at sea level (SL; 55m), 17 healthy males were transported to high altitude (HA; 4,300m) for a 22-day sojourn. AMS symptoms measured twice daily during the first 5days at HA were used to dichotomize participants according to AMS severity: moderate/severe AMS (AMS; *n*=11) or no/mild AMS (NoAMS; *n*=6). Urine samples collected on SL day 12 and HA days 1 and 18 were analyzed using proton NMR tools and the data were subjected to multivariate analyses. The SL urinary metabolite profiles were significantly different (*p*≤0.05) between AMS vs. NoAMS individuals prior to high altitude exposure. Differentially expressed metabolites included elevated levels of creatine and acetylcarnitine, and decreased levels of hypoxanthine and taurine in the AMS vs. NoAMS group. In addition, the levels of two amino acid derivatives (4-hydroxyphenylpyruvate and N-methylhistidine) and two unidentified metabolites (doublet peaks at 3.33ppm and a singlet at 8.20ppm) were significantly different between groups at SL. By HA day 18, the differences in urinary metabolites between AMS and NoAMS participants had largely resolved. Pathway analysis of these differentially expressed metabolites indicated that they directly or indirectly play a role in energy metabolism. These observations suggest that alterations in energy metabolism before high altitude exposure may contribute to AMS susceptibility at altitude. If validated in larger cohorts, these markers could inform development of a non-invasive assay to screen individuals for AMS susceptibility prior to high altitude sojourn.

## Introduction

At high altitude, hypobaric hypoxia elicits a series of physiological responses that are highly variable in humans. These responses assist in adapting to high altitude (HA; ≥2,500m) conditions, but can also lead to development of acute mountain sickness (AMS) or life-threatening forms of altitude-induced illness such as high altitude cerebral edema (HACE) and high altitude pulmonary edema (HAPE; Luks et al., 2017). Non-acclimatized AMS-susceptible individuals usually develop AMS symptoms within 6–12h after a rapid ascent and exposure to high altitudes (Bärtsch and Swenson, 2013) with symptoms generally resolving within 72h of altitude exposure (TBMED505, 2010). While self-resolving, severe AMS symptoms can be temporarily debilitating. Such effects may be an unpleasant nuisance for leisure travelers but for military personnel, AMS can compromise occupational performance.

Currently, prevention of AMS onset involves pharmaceutical and non-pharmaceutical approaches. Pharmaceutical prophylaxis has limitations as medications such as acetazolamide (Dumont et al., 2000; Kayser et al., 2008; Low et al., 2012; Luks et al., 2014) are associated with side effects that, while mild, may discourage use. Non-pharmaceutical approaches include pre-acclimatization by intermittent exposure to normobaric hypoxia (Treml et al., 2020) or spending time at moderate altitude before ascending to higher elevations (Luks et al., 2017). While pre-acclimatization carries the benefit of reducing AMS, implementation can be logistically difficult. Identifying individuals at highest risk of severe AMS before ascent would be a useful decision aid for medical preparation and planning prior to high altitude sojourn.

Besides having a prior history of AMS occurrences, there are no clinical or routine laboratory examinations that can be performed to determine AMS susceptibility. As such, there is interest in developing rapid molecular-based screening methods for that purpose. For example, Canouï-Poitrine et al. (2014) developed and validated a model intended to identify individuals at risk of developing severe AMS and other forms of altitude-induced illness (HACE and HAPE). However, the model is not amenable to widespread and easy application, as it requires subjects to undergo an exercise test regimen, while breathing a hypoxic gas mixture. Moreover, the value and accuracy of that hypoxic cardiopulmonary exercise testing model has been questioned (Bärtsch, 2014). Yang et al. (2016) suggested that serum levels of three peptides (ITIH4 347–356, ITIH1 205–214, and FGA 588–624) at sea level can be used to determine the predisposition to high altitude-induced illness. However, the effectiveness and accuracy of these peptides in predicting AMS are yet to be established, and measuring these peptides requires invasive blood collection. An ideal screening platform would be non-invasive (e.g., urine) and easy to implement.

Genetic factors have been regarded as key players in high altitude adaptation (Arestegui et al., 2011; Yu et al., 2016; Bottura et al., 2019), suggesting that genetic polymorphisms influence high altitude adaptation (Yi et al., 2010). It is possible that functional polymorphisms in key enzymes involved in physiologic pathways may drive occurrence and severity of AMS and that metabolite outputs yielded by these pathways can be determined using a metabolomics-based approach.

Metabolomics is a unique top-down approach that can be applied to study complex systems (Nicholson and Lindon, 2008). The resultant metabolite profiles are regarded as good indicators of an organism’s physiology as they measure the “end result” of multiple protein, gene, and environmental interactions (Wishart, 2007). As such, applying metabolomic approaches to examine physiological alterations resulting from altitude adaptation may not only identify biomarkers for AMS susceptibility, but may also provide further insight into the physiologic pathways affecting AMS. This exploratory effort used an untargeted metabolomics approach to identify urine metabolites that might serve as predictive markers of AMS susceptibility and provide insight into the biological pathways underpinning AMS.

## Materials and Methods

### Subjects and Study Design

The analyses reported herein used archived samples and data from a study designed to assess the efficacy of a higher protein diet for preserving fat-free mass during high altitude (HA; 4,300m) sojourn (Berryman et al., 2018). The present analyses were conceived after trial completion to explore whether measuring urine metabolites could provide insight into observed inter-individual variability in AMS severity (Karl et al., 2018a).

The study was approved by the Institutional Review Board at the United States Army Research Institute of Environmental Medicine (USARIEM) in Natick, MA and conducted May–Aug 2016. Investigators adhered to the policies for the protection of human participants as prescribed by Army Regulation 70–25, and the research was conducted in adherence with the provisions of 32 CFR Part 219. The trial is registered on https://clinicaltrials.gov/, NCT02731066.

Seventeen healthy, unacclimatized, physically active men (aged 18–42years) participated in the study. Although, study enrollment was open to both sexes, no women volunteered to participate. Study methods, and primary and secondary results have been previously reported in detail (Berryman et al., 2018; Karl et al., 2018a,b; Margolis et al., 2018; Pasiakos et al., 2018; Young et al., 2018; Hennigar et al., 2020). Briefly, the human study was a randomized, controlled trial consisting of two phases conducted over 43 consecutive days. During the 21-day first phase, participants resided at sea level (SL), consumed a self-selected weight maintaining diet, maintained habitual exercise routines, and were free living but visited the laboratory daily. On day 21, participants were flown from Boston, MA to Denver, CO, where they were placed on supplemental oxygen until arriving at the summit of Pike’s Peak, CO (4,300m) the following morning (day 0 at HA). Participants then resided for the next 21days at the United States Army Research Institute of Environmental Medicine Maher Memorial Laboratory, Pike’s Peak, CO (phase 2; HA). During HA, participants were under constant supervision, consumed a controlled and measured diet, and engaged in prescribed physical activity. Participants started consuming controlled diets on day 1, the first full day of residence at 4,300m, and continued until the end of the sojourn at HA (phase 2). Diets contained either a standard amount of protein (1.1g/kg/day; *n*=8) or higher amount of protein (2.1g/kg/day; *n*=9), and were designed to induce weight loss, which is common during military training and operations, and during HA sojourn (Hamad and Travis, 2006).

The prevalence and severity of AMS was assessed using the shortened version of the Environmental Symptoms Questionnaire (ESQ; Beidleman et al., 2007). The ESQ was administered twice daily during the first 5days at HA and used to calculate AMS weighted cerebral factor scores (Beidleman et al., 2007). Peak scores were recorded from all participants during the first 48h at HA, and were used to categorize AMS severity as mild (≥0.7 and <1.53), moderate (≥1.53 and <2.63), and severe (≥2.63; Beidleman et al., 2013; Karl et al., 2018a). The analyses described below used two group identifiers: AMS and NoAMS. For the purposes of this analysis, subjects who scored less than 1.53 were designated as belonging to the NoAMS (no/mild AMS symptoms) group (*n*=6), while those with scores greater than 1.53 were designated as the AMS (moderate/severe AMS symptoms) group (*n*=11).

### Urine Sample Collection and Preparation

Urine samples obtained at sea level 9days prior to accent to altitude (SL) and at high altitude on days 1 (HA1) and 18 (HA2) were used for the analyses described herein. Collections began at 0730 on SL day 12 and 0700 on HA days 1 and 18 following an overnight fast and required participants to collect all urine produced over 2h. During that 2-h period, participants consumed their individualized standard or higher protein breakfast on all 3 test days. Day 12 was the only day participants ate their diet group-specific breakfast during SL. Aliquots of the SL samples were frozen ^1^H nuclear magnetic resonance (NMR) metabolomic analyses and shipped to Wright Patterson Air Force Base (WPAFB), Dayton, OH for analyses. Upon arrival at WPAFB, all urine samples were stored at −80°C. The preparation of urine samples for ^1^H NMR spectral data acquisition followed the procedure described in Sibomana et al. (2017).

### NMR Data Acquisition and Processing

All proton NMR spectra were acquired using a Varian INOVA NMR instrument operating at 600MHz and a probe temperature of 25°C. NMR spectral data acquisition and processing are routinely performed in our laboratory. These procedures were conducted as previously described (Sibomana et al., 2017).

### NMR Data Analyses

Multivariate data analyses were conducted on binned and scaled spectral data. Binned NMR data were scaled to the entire dataset chosen as reference by subtracting each bin value from the mean value for the corresponding bin in the reference data (whole dataset), then dividing this value by the SD of the reference data (auto-scaling).

#### Unsupervised Analysis

Principal Component Analysis (PCA) was used as an unsupervised analysis technique and provided a first approach for data visualization (Mahle et al., 2011). The PCA model was constructed based on the data for AMS group at SL and HA1. Data for the AMS group at HA2 and NoAMS group at SL, HA1, and HA2 were then superimposed onto the PCA scores plot. The quality of data clustering in this PCA model was evaluated using Davies-Bouldin (Davies and Bouldin, 1979) and Silhouette (Rousseeuw, 1987) indexes. Davies-Bouldin index (DBI) is defined as a ratio between the within group distances (intra group scatter) and the between group distances (group’s separation). The lower the DBI value, the better the cluster separation and the tightness inside the groups. The silhouette value is a measure of how similar a data point is to its own cluster (group) compared to other clusters (groups). The higher the value, the better the data point matches to its own cluster (group) and the poorer it matches to neighboring clusters (groups).

#### Supervised Analysis

Orthogonal Projection onto Latent Structures – Discriminant Analysis (OPLS-DA) was used as a supervised technique to classify data and identify salient features that allow class separation of AMS vs. NoAMS at SL, HA1, and HA2 (Wold et al., 2001). The *Q*^2^ (coefficient of prediction) metric was used to evaluate the predictive ability of the OPLS models as described in report of Sibomana et al. (2017). A permutation test was also performed to evaluate the significance of the *Q*^2^ metric. This test involved repeatedly permuting the data labels and re-running the discrimination analysis, resulting in a distribution of the *Q*^2^ scores (Westerhuis et al., 2008). The *Q*^2^ from the correctly labeled data is then compared to the distribution to determine the significance of the model at a specified alpha (set herein as *α*=0.01). A receiver operator characteristic (ROC) curve was also used as a secondary validation of an OPLS binary model and the area under the curve (AUC) was calculated. Evaluation of the significance of this AUC value was conducted using the same permutation procedure as described above.

The variable selection (salient bins) from OPLS-DA was statistically evaluated as described in Sibomana et al. (2017). Briefly, the bin loadings, commonly referred to as coefficients, were compared to calculated null distributions in order to select for significance. The null distribution for each bin was determined by refitting the OPLS model to datasets, in which each bin was independently and randomly permuted to remove any correlation between it and AMS/NoAMS groups. The true OPLS model loading was then compared to the resulting null distribution of loadings, and values in the tail (greater than 99.5% or less than 0.5% of the null distribution; corresponding to *α*=0.01) were assumed to contribute significantly to the model. The permutation was initially repeated 1,000 times for each bin and those near-significant loadings (greater than 92.5% or less than 7.5% of the null distribution; corresponding to *α*=0.2) were selected for 500 additional permutations (total 1,500).

### Quantification of Metabolite Resonances

Normalized NMR spectra (PQN method; see above) were used to quantify metabolite resonances determined to be important for group classification. Quantification of specific metabolite resonances was accomplished using an interactive spectral deconvolution algorithm in MATLAB adapted from Anderson et al. (2011). The deconvolution tool fits a defined spectral region using a combination of tunable baseline shapes (spline, v-shaped, linear, or constant) and a Gauss-Lorentz peak-fitting function. Metabolite peak intensities (total peak area) represent a semi-quantitative assessment of urine metabolites since this biofluid accumulates in the bladder over a variable period of time (i.e., 8h) and its volume cannot be controlled. Although, the PQN method of spectral normalization helps to adjust for variable urine concentrations, absolute quantitative amounts of each metabolite are not reported. However, the semi-quantitative metabolite measurements reported herein do allow a relative comparison between samples.

Nuclear magnetic resonance spectral regions identified as significant by OPLS-DA were compared between time points (SL, HA1, and HA2) and AMS vs. NoAMS, and specific resonances were assigned to metabolites with the aid of literature, on-line databases (HMDB, http://www.hmdb.ca/, www.bmrb.wisc.edu, etc.), and by “spiking” samples with known compounds, if necessary. Signal intensities were integrated to obtain relative measures of metabolite concentrations at each time point.

### Creatine Assays

Creatine assays were performed on additional archived urine samples collected at the same time as samples used for the NMR analysis. Assays were conducted using an Abcam (Cambridge, United Kingdom) creatine activity assay kit (ab65339) according to manufacturer instructions.

### Statistical Analyses

A repeated measures MANOVA was conducted to examine effects of time and AMS status (AMS vs. NoAMS) on urine metabolite profiles. Only metabolites identified in OPLS-DA as significant were subjected to MANOVA. For metabolites demonstrating time-by-AMS group interactions (*p*<0.05), Levene’s and Welch’s tests were conducted to assess the equality of variances between the data for SL, HA1, and HA2 or AMS vs. NoAMS groups for each metabolite using statistical software package JMP® 11.0.0 (SAS Institute, Cary, NC, United States). If Levene’s test was significant (*p*≤0.05), then a Welch’s nonparametric ANOVA test was used to determine if there were significant differences in the mean values between groups for the metabolite of interest. If the Levene’s test was not significant, significance was tested using a one-way ANOVA (*t*-test). If both Levene’s and Welch’s tests were significant (*p*≤0.05), a pairwise Welch test was performed for all pairs of groups. No false discovery rate correction was applied to the data since OPLS-DA and MANOVA were used to down-select metabolites. Only metabolites identified by both data analysis methods were considered as statistically significant. Results are expressed as mean±SEM and are considered statistically significant at *p*≤0.05. Cohen’s d (effect size; Cohen, 1988) was used as a measure of the magnitude of changes in the level of each urinary metabolite noted at HA1 and HA2 relative to SL by subtracting the value obtained for SL from those obtained for HA (HA1 or HA2) and assessing the difference relative to the pooled SDs for HA (HA1 or HA2) and SL.

## Results

### Urinary Metabolite Alterations Over Time

**Figure 1:**
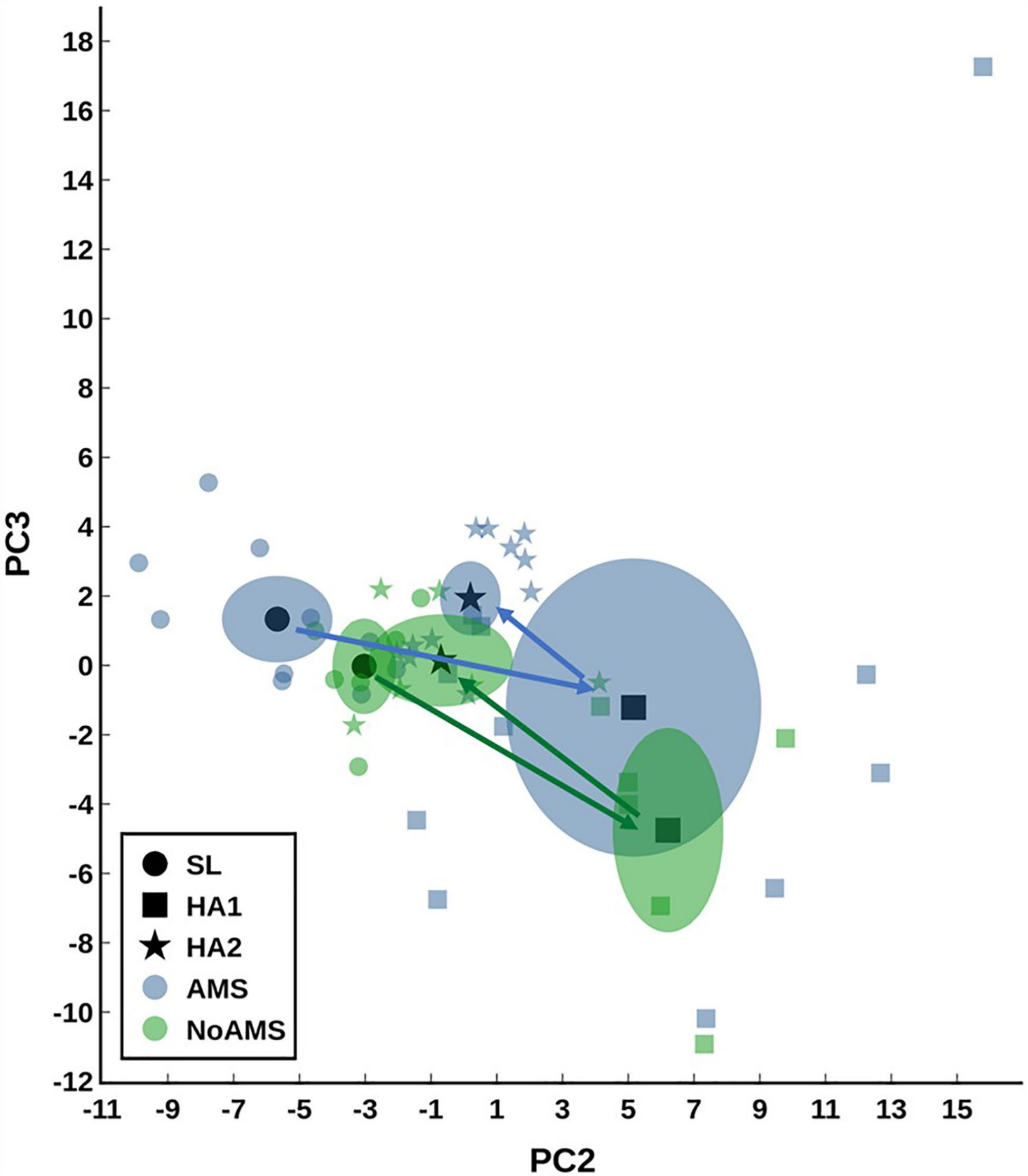
Principal component analysis (PCA) scores plot modeling the urine data for acute mountain sickness (AMS) group at sea level (SL) and high altitude (HA) at Day 1 (HA1). Data for AMS group at high altitude day 18 (HA2) and those for no/mild AMS (NoAMS) group at SL, HA1, and HA2 are superimposed in the model. Data were autoscaled using all groups as reference. Data are plotted showing the centroid mean±2 SE (or 95% CI) as well as the individual data points. The arrows show the trajectory from SL to HA1 to HA2.

The mean peak AMS-weighted cerebral factor score for AMS individuals (2.25±0.18; *n*=11) was significantly elevated (*p*<0.05) compared to in NoAMS subjects (0.78±0.18; *n*=6). AMS severity (i.e., NoAMS vs. AMS) was unrelated to diet group (Karl et al., 2018a). PCA analysis indicated that the urinary metabolite profiles for both groups changed over the time course of the study with the AMS group displaying greater variation in data at HA1 compared to NoAMS (Figure 1). PCA also clearly separated urinary profiles for AMS from NoAMS at all time points, with differences being most apparent at SL (Figure 1). The urinary profiles for both groups at HA2 indicated a trajectory returning toward SL. Mapping positions for NoAMS at SL and at HA2 partially overlapped, indicating some similarities in metabolite profiles. In contrast, there is a clear separation in profiles within the AMS group at these two time points.

**Figure 2:**
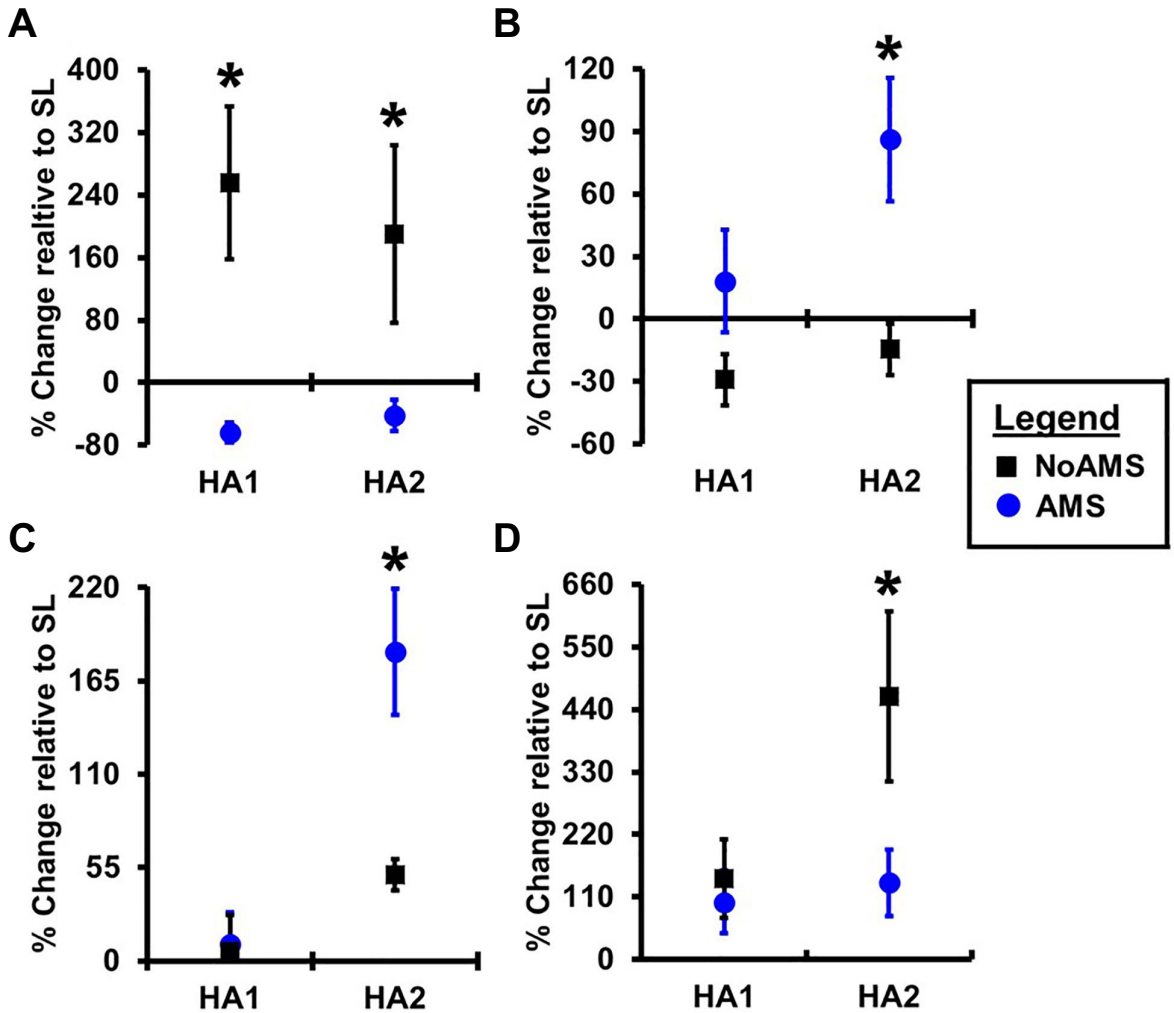
Percent change in peak intensity of **(A)** creatine, **(B)** N-methylhistidine, **(C)** hypoxanthine, and **(D)** acetylcarnitine 4,300m on day 1 (HA1) and day 18 (HA2) relative to SL for urine samples collected from AMS susceptible (blue symbol) and NoAMS resistant subjects (black symbol). Asterisk denotes significant difference between groups (*p*≤0.05).

Changes in urinary metabolite levels that occurred from SL to HA1 and SL to HA2 indicated that changes in the levels of only four metabolites differed between AMS or No AMS groups (Figure 2). These metabolites included creatine (energy metabolism), two amino acid derivatives consisting of N-methylhistidine and acetylcarnitine, and hypoxanthine (nucleotide derivative). As shown in Figure 2A, urinary creatine levels decreased 64% from SL to HA1 in the AMS group, but increased by 256% in the NoAMS group. At HA2, creatine levels were 42% lower than that noted at SL in the AMS group, but increased by 190% from SL to HA2 in the NoAMS group. The levels of hypoxanthine (Figure 2C) and acetylcarnitine (Figure 2D) increased by 182 and 135%, respectively, from SL to HA1 for the AMS group, while in the NoAMS group, levels increased by 51 and 463%, respectively. At HA2, N-methylhistidine levels (Figure 2B) for AMS increased by 86% relative to SL, while NoAMS subjects decreased by 15%. It is noteworthy to indicate that creatine, hypoxanthine, and N-methylhistidine were among the metabolite classifiers of these two groups at SL.

### Urinary Metabolites at Sea Level Compared to AMS Incidence

**Figure 3:**
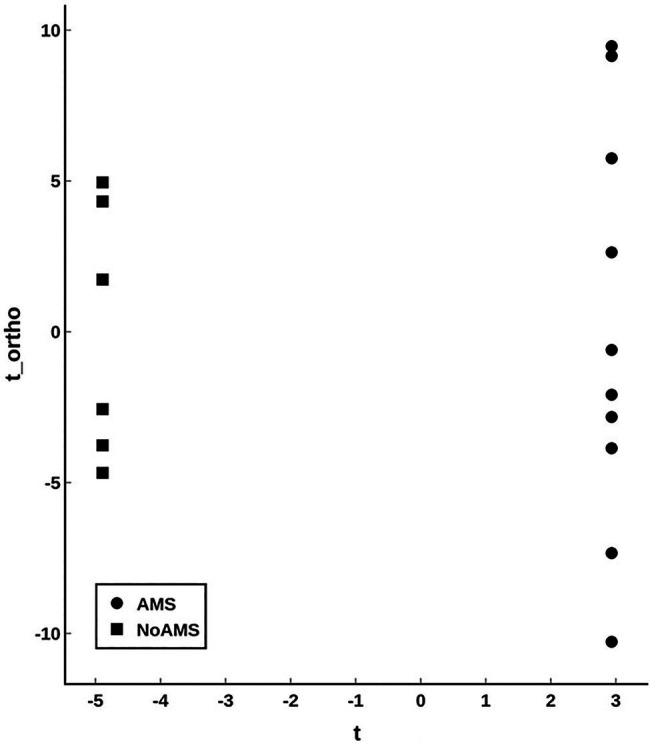
Orthogonal projection onto latent structures – discriminant analysis (OPLS-DA; T-scores plot) modeling the urinary metabolite data for AMS (solid circle) vs. NoAMS (solid square) groups at SL. Data were autoscaled using all groups as reference. The analysis is highly significant with *Q*^2^=1.0 (*p*<0.001).

**Figure 4:**
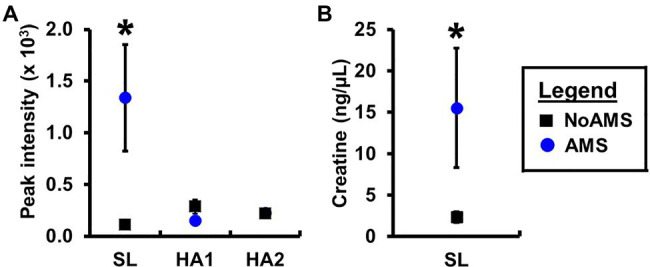
Urinary creatine levels for AMS susceptible (blue symbol) and NoAMS resistant subjects (black symbol) at SL, and 4,300m on day 1 (HA1) and day 18 (HA2) determined using the **(A)** ^1^H NMR technique and **(B)** creatine assay. Asterisk denotes significant difference between groups (*p*≤0.05).

**Figure 5:**
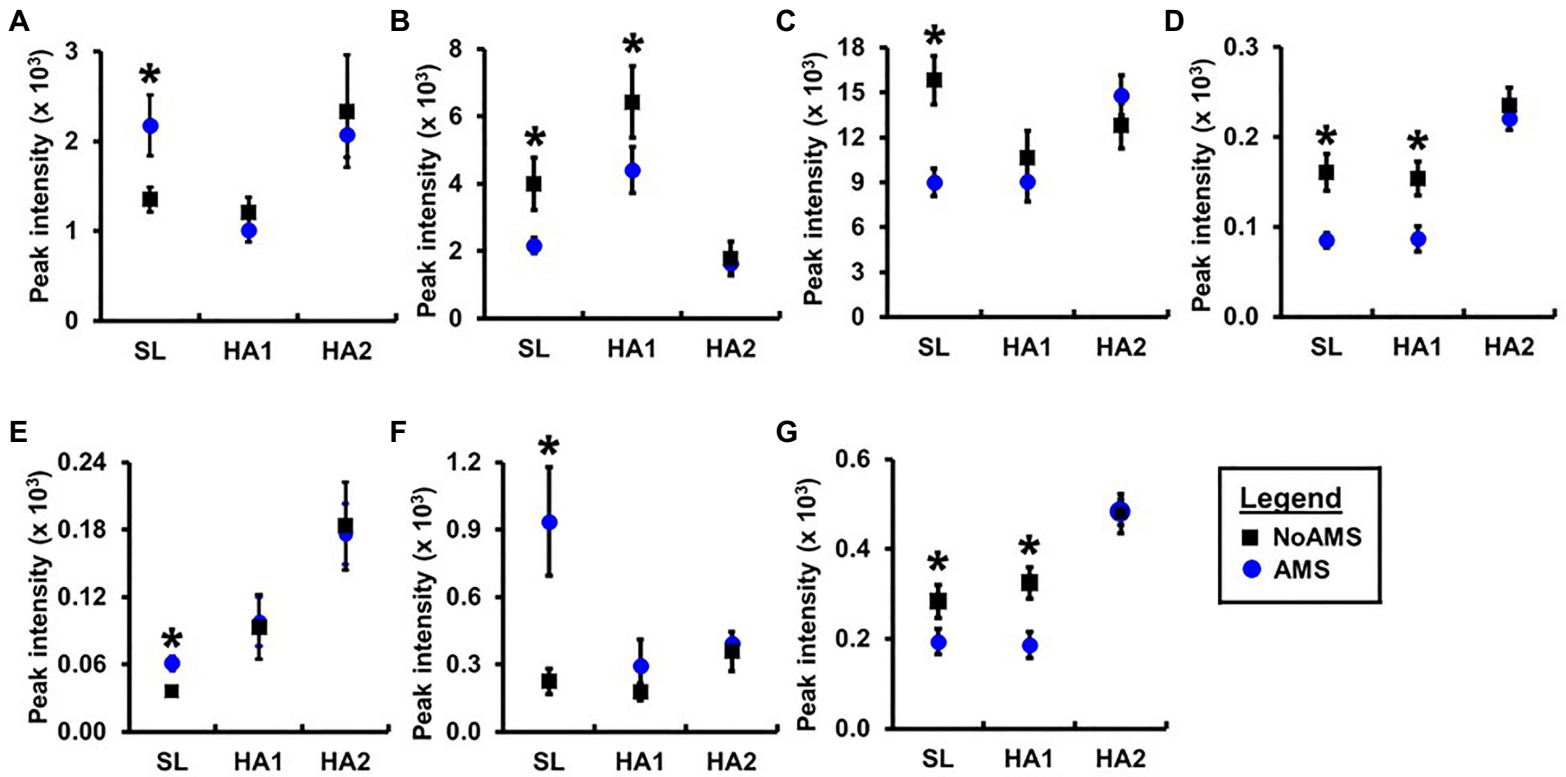
Relative peak intensities of **(A)** 4-hydroxyphenylpyruvate, **(B)** taurine, **(C)** N-methylhistidine, **(D)** hypoxanthine, **(E)** acetylcarnitine, unidentified peaks at **(F)** 3.33ppm (doublet) and **(G)** 8.20ppm (singlet) in 1H nuclear magnetic resonance (NMR) spectral data for urine samples collected from AMS susceptible (blue symbol) and NoAMS resistant subjects (black symbol) at SL, and 4,300m on day 1 (HA1) and day 18 (HA2). Asterisk denotes significant difference between groups (*p*≤0.05).

Orthogonal projection onto latent structures – discriminant analysis comparing AMS and NoAMS at SL (Figure 3) yielded a *Q*^2^ value of 1.0 (*p*=0.001), a predictive accuracy of 100% (leave-1-out cross validation) and an AUC value of 1.0 (*p*=0.001). Further, the T-score scatter plots of the data confirmed that the urine metabolite profiles for the AMS group clustered together and were separated from the NoAMS group. Examination of metabolites that classified these two groups at SL indicated that creatine was the strongest driver of separation between the two groups (Figure 4). Spectra from the ^1^H NMR analysis showed that AMS subjects had (*p*≤0.05) higher relative peak intensity for creatine at SL (1.34±0.52) as compared to NoAMS subjects (0.11±0.03; Figure 4A). These observations were confirmed by a secondary method using creatine assay analyses (Figure 4B). Additional metabolites driving discrimination between groups at SL included 4-hydroxyphenylpyruvate, taurine, N-methylhistidine, acetylcarnitine, hypoxanthine, and two unidentified metabolites (Figure 5). Urinary excretion levels of taurine (Figure 5B), N-methylhistidine (Figure 5C), hypoxanthine (Figure 5D), and one unidentified metabolite (a singlet at 8.20ppm; Figure 5G) were lower in AMS vs. NoAMS at SL, while the levels of 4-hydroxyphenylpyruvate (Figure 5A), acetylcarnitine (Figure 5E), and unidentified metabolite at 3.33ppm (doublet; Figure 5F) were elevated in the AMS group. The levels of taurine, hypoxanthine, and unidentified metabolite at 8.20ppm for AMS subjects were still lower than values obtained for the NoAMS group at HA1, but these differences between groups disappeared at HA2.

## Discussion

### Changes in Urinary Metabolite Profiles Over Time

Principal component analysis results indicated that the urinary metabolite profiles for AMS and NoAMS groups changed significantly as the subjects moved from SL to HA and during their stay at altitude, reflecting the subject’s response to altitude environment. The changes in metabolite profiles from SL to HA reflect alterations in metabolic pathways, which are likely driven by complex adaptive changes in multiple biological systems responding to hypobaric hypoxia. The AMS group displayed greater variation in data at HA1 (Figure 1) compared to NoAMS group, highlighting AMS subject’s diverse responses to high altitude conditions. The observations that metabolite profiles for both groups were distinct at SL and became more similar at high altitude, suggest the existence of the urinary metabolite signatures for AMS susceptibility that may be apparent before exposure to altitude-induced stress. Thus, the discussion focuses on the metabolite differences between AMS and NoAMS at SL. Eight urinary metabolites that separated AMS from NoAMS individuals at sea level were identified. These metabolites included creatine, hypoxanthine, taurine, acetylcarnitine, N-methylhistidine, 4-HPPA, and two unknowns (Figures 4, 5).

### Cellular Availability of Creatine and Hypoxantine in AMS Susceptible Subjects

Of the metabolite alterations seen at sea level, creatine had the highest contribution to the PCA segregation of NoAMS subjects. The average urinary creatine level in AMS susceptible individuals was 12-fold greater at sea level than NoAMS subjects (Figure 4). This difference could result from one or more factors including: (1) a higher dietary intake of creatine-containing foods, (2) a lower conversion rate of creatine to phosphocreatine and creatinine, and (3) decreased cellular retention of creatine. In this study, dietary protein intake did not differ between groups at sea level and volunteers reported compliance with instructions not to consume any supplements. While urinary phosphocreatine excretion was not examined, urine creatinine levels did not differ between groups. Therefore, we hypothesize that the simplest and most likely explanation for the higher creatine excretion rate in AMS individuals is decreased cellular retention.

Lower creatine cellular retention at sea level would lead to an increased rate of urinary elimination, limiting cellular availability of the substrate required for phosphocreatine synthesis once shifted to hypoxic conditions. The implication is that in AMS subjects, cells may have an existing deficiency in an energy supply needed to cope with altitude-induced hypoxia. Hypoxia is known to affect cellular ATP production through downregulation of several tricarboxylic cycle enzymes (Green et al., 1989; Howald et al., 1990; Levett et al., 2012, 2015; Murray and Horscroft, 2016) as well as compromising electron transport chain complexes (Howald et al., 1990; Levett et al., 2012, 2015; Colleoni et al., 2013; Murray and Horscroft, 2016). Indeed, several studies also suggest that lower cellular creatine levels increase sensitivity to hypoxia (Wilken et al., 1996; Turner et al., 2015; Scheer et al., 2016). In contrast, Turner et al. (2015) reported that hypoxia-induced decrements in a wide range of neuropsychological measures were corrected by creatine supplementation. Other studies suggest that stored phosphocreatine may play a significant role in sustaining synaptic transmission during hypoxia (Lipton and Whittingham, 1982), and that creatine supplementation can enhance the cellular adaptive response to hypoxia mediated by HIF-1 in cardiomyocytes (Santacruz et al., 2017). Collectively, these findings suggest that cellular creatine availability is critical to sustaining intracellular phosphocreatine and ATP pools during hypoxic conditions. Our findings of increased urinary excretion of creatine at sea level in AMS subjects suggest that existing deficiencies of cellular creatine levels may increase hypoxia sensitivity.

**Figure 6:**
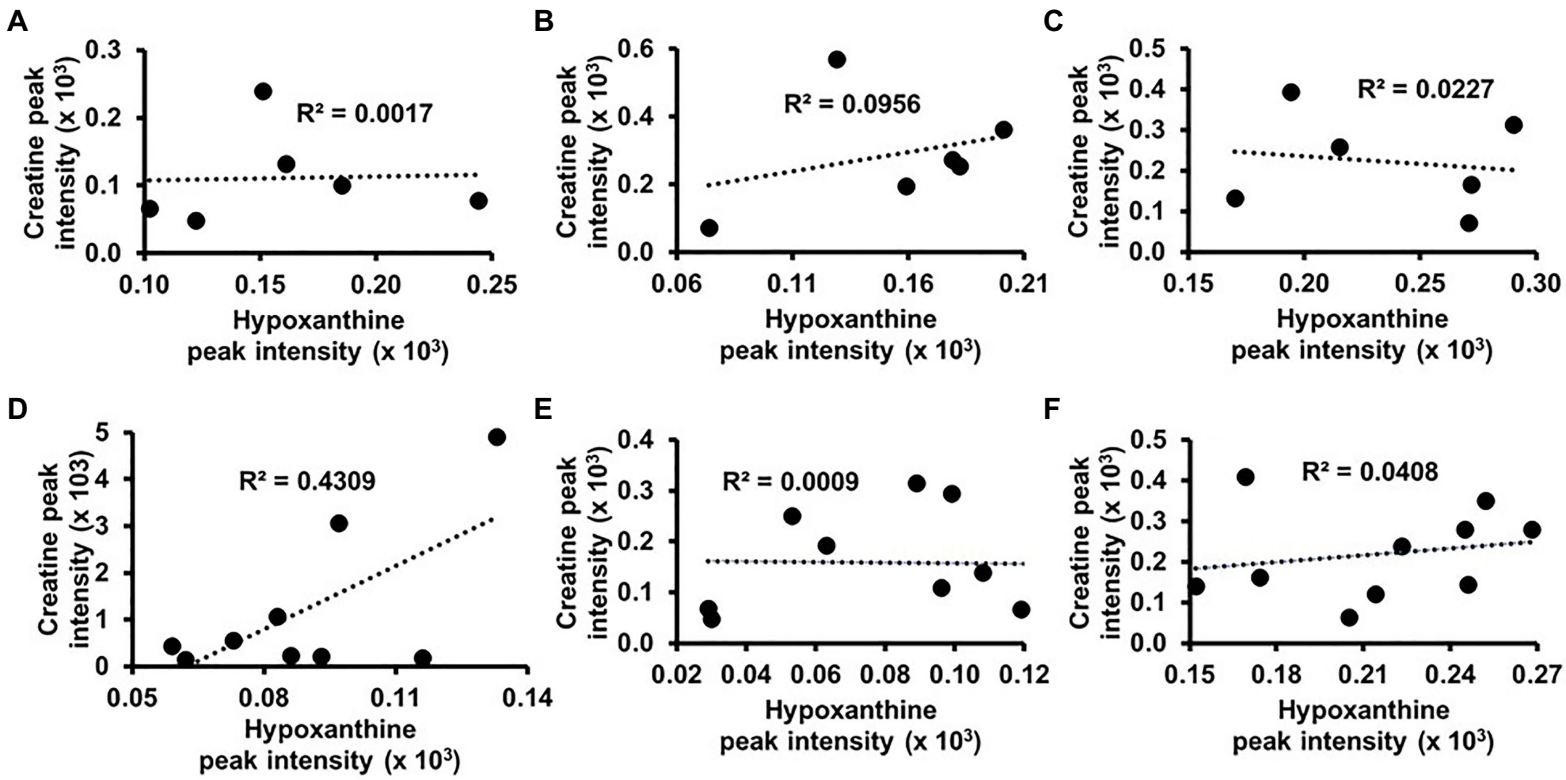
Correlation analyses between urinary levels of creatine and hypoxanthine for NoAMS subjects at **(A)** SL, **(B)** 4,300m on day 1 (HA1), **(C)** 4,300m on day 18 (HA2), and AMS-susceptible individuals at **(D)** SL, **(E)** HA1, and **(F)** HA2.

Hypoxanthine was also among the metabolites that classified AMS and NoAMS groups at SL (Figure 5D). Hypoxanthine is a naturally occurring purine degradation by-product, and cellular levels are associated with cellular levels of creatine. For example, hypoxanthine supplementation has been shown to reverse hypoxia-induced depletion of cellular creatine and phosphocreatine pools (Lee et al., 2018). Findings of the present study suggest that cellular levels of hypoxanthine may be lower in AMS subjects which could, in turn, impair the cellular retention of creatine and account for its higher urinary excretion. While a correlation analysis using the ^1^H NMR urinary creatine and hypoxanthine data did not indicate a creatine/hypoxanthine correlation for NoAMS individuals (Figures 6A–C), data for AMS individuals demonstrated a positive relationship between these two metabolites at SL (*R*^2^=0.4309; Figure 6D) but not at HA1 (Figure 6E) or HA2 (Figure 6F).

### Other Urinary Metabolite Differences Seen in Acute Mountain Sickness Susceptibility

Acute mountain sickness subjects also demonstrated significantly lower taurine excretion at sea level and Day 1 at altitude relative to NoAMS individuals. Of note, previous studies have suggested that this biogenic amine plays a significant role in protecting cells against hypoxia-induced damage (Crass and Lombardini, 1977; Franconi et al., 1985; Malcangio et al., 1989; Michalk et al., 1997; Amano et al., 2003; Chen et al., 2009, 2013). Further, under hypoxic conditions, taurine supplementation has been shown to improve cardiovascular function in pigs (Franconi et al., 1985), attenuate vascular remodeling in rats (Amano et al., 2003), and prevent learning impairment and increase survival time in mice (Malcangio et al., 1989). Although, taurine’s mechanisms of protection against hypoxia-mediated decrements are not well understood, taurine may act as a potent endogenous agent to induce cellular growth despite oxygen deficiency, and improve both osmotic status and calcium homeostasis (Michalk et al., 1997). Collectively, these findings suggest that taurine may play an important role in counteracting hypoxic-induced cellular damage. The lower urinary excretion of taurine seen at sea level and Day 1 at altitude in AMS subjects may reflect an increase in degradation of this metabolite. Unfortunately, the current study did not investigate the taurine catabolism pathway.

Acetylcarnitine plays a critical role in cellular energy metabolism and has been shown to play a role in cellular responses to hypoxia-induced stress (Aureli et al., 1994; Scafidi et al., 2010). Barhwal et al. (2007) demonstrated that daily supplementation of acetylcarnitine to rats during hypoxic exposure ameliorated hypoxia-induced deficits in spatial working memory, oxidative stress, and apoptotic cascades, suggesting that this metabolite plays a significant role in the body’s response to hypoxic stress. In the current study, urinary acetylcarnitine excretion in AMS susceptible individuals was higher than for NoAMS individuals at SL (Figure 5E). This may suggest that the cellular stores of this metabolite were lower in AMS individuals, and their increased susceptibility to AMS may be mediated by alteration in energy or lipid metabolism.

Urinary N-methylhistidine is formed in the body through methylation of peptide-bound histidine in muscle actin and myosin and eliminated in urine after protein breakdown (Long et al., 1975). Urinary excretion of N-methylhistidine is regarded as useful indicator for muscle protein breakdown provided that the individual has a meat-free diet (Munro and Young, 1978; Tomas et al., 1979; Elia et al., 1980, 1981). Though dietary protein can affect urinary excretion (Omstedt et al., 1978), it is unlikely in this study that the diet was driving the lower N-methylhistidine in AMS vs. NoAMS as dietary protein intake did not differ at SL. Of note, previous studies have shown that the levels of N-methylhistidine are altered in individuals sensitive to high altitude. For example, plasma levels of methylhistidine have previously been shown to increase in patients with HAPE compared to controls (Luo et al., 2012). That report conflicts with our findings on urinary levels of N-methylhistidine. However, it is possible that the disparity between the two studies derives from differences in the test matrices (i.e., urine vs. blood) used.

Finally, increased urinary excretion at sea level of 4-HPPA in AMS individuals suggest a pre-existing alteration in the phenylalanine catabolism pathway and/or 4-HPPA degradation pathway may contribute to AMS susceptibility. However, phenylalanine and tyrosine levels in the urine were not statistically different between groups. As the downstream of 4-HPPA degradation pathway was not investigated in this study, a more thorough study examining the molecular mechanisms for excessive 4-HPPA urinary elimination awaits future efforts.

### Study Limitations and Future Study Modifications

In the current study, the diet was not a controlled variable at sea level (except on day 12) and day 0 at high altitude prior to starting the study diet regimen. As such, further studies are needed to investigate whether potential dietary factors can exert significant impact on the individual’s susceptibility or resistance to AMS and should include a control group with controlled dietary input throughout the study, at sea level as well as at altitude. In addition, the sample size was limited to *n*=11 and *n*=6 for AMS and NoAMS groups, respectively. Follow-up studies using a larger sample sizes will be required to validate the biomarker potential as described here. In addition, the subjects spent 21days at SL and 22days at HA with sample collection limited to one time at SL (Day 12) and two times at altitude (Day 1 and Day 18). Since there was a considerable amount of time elapsing between sample collections at altitude, the sampling scheme did not capture temporal changes that may have transpired, especially during the 0–72h period at altitude when the greatest molecular changes are expected to occur. Future studies should also consider additional sample collection time points at SL to establish a firm and consistent baseline. Lastly, female subjects should also be included in future efforts to allow examination of gender-related responsivity and, possibly, unique gender-based metabolite signatures.

## Conclusion

This study identified a set of eight urinary metabolites using NMR-based metabolomics that, at sea level prior to altitude exposures, discriminated individuals who later experienced more severe acute mountain sickness upon ascent to high altitude. Urinary creatine, hypoxanthine, acetylcarnitine, 4-HPPA, N-methylhistidine, and taurine were among the classifiers of acute mountain sickness sensitive individuals. The observed metabolite differences between AMS and NoAMS at sea level reflect modulations in metabolic pathways that may result from genetic differences and other interacting factors. However, an examination of literature suggests that the urinary levels of these metabolites, directly or indirectly, play a role in energy metabolism and have been shown in other studies to influence physiologic responses to hypoxia in *in vivo* and *in vitro* models.

These results suggest that a specific set of urinary metabolites could potentially be used to identify AMS susceptible subjects even before exposure to altitude and exhibition of altitude-mediated sickness symptoms. If biological plausibility can be confirmed and findings validated in larger cohorts, these metabolites may comprise a metabolic biomarker signature that can potentially be used to non-invasively screen individuals for vulnerability to altitude-induced illness. In addition to biomarker development, these metabolites may provide insight into specific mechanisms involved in the pathophysiological process of AMS. This information would be of importance in designing new individualized approaches and therapeutics that can prevent or attenuate the impact of AMS.

## Data Availability Statement

The raw data supporting the conclusions of this article will be made available by the authors, without undue reservation.

## Ethics Statement

The studies involving human participants were reviewed and approved by the Institutional Review Board at the U.S. Army Research Institute of Environmental Medicine (USARIEM) in Natick, MA. The patients/participants provided their written informed consent to participate in this study.

## Author Contributions

IS: metabolomics methodology, formal analysis, investigation, resources, data curation, writing – original draft, writing – review and editing, and visualization. DF and MR: metabolomics formal analysis. NR: metabolomics formal analysis and writing – review and editing. JK: human study conceptualization, methodology, formal analysis, investigation, resources, data curation, writing – review and editing, visualization, supervision, and project administration. CB: human study conceptualization and analysis, methodology, formal analysis, investigation, data curation, writing – review and editing, and project administration. AY: human study conceptualization, methodology, investigation, supervision, and writing – review and editing. SP: human study conceptualization, methodology, resources, investigation, supervision, and writing – review and editing. CM: metabolomics formal analysis, investigation, resources, data curation, writing – original draft, writing – review and editing, visualization, supervision, and project management. All authors contributed to the article and approved the submitted version.

## Funding

This work was supported by the United States Army Medical Research and Development Command and the United States Department of Defense, Defense Health Program.

## Conflict of Interest

The authors declare that the research was conducted in the absence of any commercial or financial relationships that could be construed as a potential conflict of interest.

## Publisher’s Note

All claims expressed in this article are solely those of the authors and do not necessarily represent those of their affiliated organizations, or those of the publisher, the editors and the reviewers. Any product that may be evaluated in this article, or claim that may be made by its manufacturer, is not guaranteed or endorsed by the publisher.
